# The longitudinal relationship of changes of adiposity to changes in pulmonary function and risk of asthma in a general adult population

**DOI:** 10.1186/1471-2466-14-208

**Published:** 2014-12-22

**Authors:** Runa V Fenger, Arturo Gonzalez-Quintela, Carmen Vidal, Lise-Lotte Husemoen, Tea Skaaby, Betina H Thuesen, Mette Aadahl, Flemming Madsen, Allan Linneberg

**Affiliations:** Research Centre for Prevention and Health, The Capital Region of Denmark, Building 84-85, Nordre Ringvej 57, DK-2600 Glostrup, Denmark; Department of Medicine, Complejo Hospitalario Universitario, Santiago de Compostella, Spain; The Danish Lung function Laboratory, Helsingør, Denmark; Department of Clinical Experimental Research, Glostrup University Hospital, Glostrup, Denmark; Department of Clinical Medicine, Faculty of Health and Medical Sciences, University of Copenhagen, Copenhagen, Denmark

**Keywords:** Adiposity, Asthma, Atopy, F_E_NO, Longitudinal, Lung function, Obesity

## Abstract

**Background:**

Adiposity has been linked to both higher risk of asthma and reduced lung function. The effects of adiposity on asthma may depend on both atopic status and gender, while the relationship is less clear with respect to lung function. This study aimed to explore longitudinal weight changes to changes in forced expiratory volume in first second (FEV1) and forced vital capacity (FVC), as well as to incident cases of asthma and wheezing, according to atopy and gender.

**Methods:**

A general population sample aged 19–72 years was examined with the same methodology five years apart. Longitudinal changes in weight, body mass index, waist circumference, and fat percentage (bio-impedance) were analyzed with respect to changes of FEV1 and FVC (spirometry), and incidence of asthma and wheezing (questionnaire). Gender, atopy (serum specific IgE-positivity to inhalant allergens) and adipose tissue mass prior to adiposity changes were examined as potential effect modifiers.

**Results:**

A total of 2,308 persons participated in both baseline and five-year follow-up examinations. Over the entire span of adiposity changes, adiposity gain was associated with decreasing levels of lung function, whereas adiposity loss was associated with increasing levels of lung function. All associations were dependent on gender (p-interactions < 0.0001). For one standard deviation weight gain or weight loss, FEV1 changed with (+/−)72 ml (66-78 ml) and FVC with (+/−)103 ml (94-112 ml) in males. In females FEV1 changed with (+/−) 27 ml (22-32 ml) and FVC with (+/−) 36 ml (28-44 ml). There were no changes in the FEV1/FVC-ratio. The effect of adiposity changes increased with the level of adipose tissue mass at the start of the study (baseline), thus, indicating an aggregate effect of the total adipose tissue mass. Atopy did not modify these associations. There were no statistically significant associations between changes in adiposity measures and risk of incident asthma or wheeze.

**Conclusions:**

Over a five-year period, increasing adiposity was associated with decreasing lung function, whereas decreasing adiposity was associated with increasing lung function. This effect was significantly greater in males than in females and increased with pre-existing adiposity, but was independent of atopy.

**Electronic supplementary material:**

The online version of this article (doi:10.1186/1471-2466-14-208) contains supplementary material, which is available to authorized users.

## Background

Adiposity has consistently been associated with asthma [1] and has also been associated with impaired lung function, e.g. as assessed by decreased levels of forced expiratory volume in first second (FEV1) and forced vital capacity (FVC) [2–6]. Thus, adiposity may influence the lungs in several ways, e.g. through inflammation leading to asthma-like, obstructive changes, or in terms of a mechanical impact on lung function (restrictive changes).

Adiposity increases the risk of asthma and may cause more severe symptoms along with a reduced response to medications [1, 7, 8]. Some studies have indicated that these effects are stronger in non-atopic individuals than atopic individuals [9–11], although contrary results also have been noted [12]. Further, adiposity has been reported to be more strongly associated with asthma types characterised by non-eosinophilic inflammation rather than by eosinophilic inflammation [13]. However, it has not been investigated whether adiposity also has differential effects on lung function in atopic and non-atopic individuals or in individuals with or without eosinophilic inflammation in the airways. Underlying pathological processes, such as eosinophilic or neutrophilic inflammation [14], could possibly modify the longitudinal relationship of adiposity with lung function.

Mechanically, adiposity may cause an extra load on the thoracic cage [15], increase the intra-abdominal pressure, and impede the movements of the diaphragm [16]. This has in smaller experimental settings been reported to decrease static lung volumes and lead to breathing at smaller tidal volumes [17]. Cross-sectional studies have suggested that these changes are stronger in persons with predominantly abdominal adiposity than in persons with predominantly general adiposity [18]. Yet, prospective population based studies are sparse and have assessed adiposity changes mainly by weight or BMI [2, 3, 19–22], except from one study that included waist and hip circumferences but not FVC [4]. Therefore, studies quantifying longitudinal changes of lung function with respect to different adiposity phenotypes may add further information about the longitudinal relationship between adiposity and lung function.

This prospective general population study aimed to investigate the association of longitudinal changes in weight, body mass index (BMI), waist circumference (WC), and fat percentage with longitudinal changes in FEV1 and FVC, and with concomitant incidence of asthma and wheezing. Further, we examined whether these associations were modified by gender, atopy, or eosinophilic inflammation of the lower airways as reflected by forced expiratory nitric oxide (F_E_NO).

## Methods

### Study population

The current study used already sampled data from the Health2006 study and the Health2006-follow-up study conducted five years apart. The participants in the baseline Health2006 cohort were drawn as a random sample from the background population aged 18–69 years, living in 11 municipalities in the south-western part of suburban Copenhagen. A total of 3471 individuals (44.7%) entered the study and participated in the health examination, which took place in 2006–08 (Health2006 baseline [23]). In 2011–12, participants in the baseline Health2006 were invited for a 5-year follow-up examination including essentially the same study protocol. A total of 3,405 were eligible for invitation (21 had emigrated and 45 died). A total of 2,308 (68.6%) agreed to participate and were re-examined between November 2011 and November 2012. Fourteen participants did not have valid measures of either FEV1 or FVC at either baseline or follow-up and were excluded from the study, which for the main analyses was based on 2,294 participants. All participants gave written informed consent before taking part in the study, which was approved by the ethics committee of the Capital Region of Denmark, Copenhagen (KA20060011).

### Questionnaire data

Information on socio-demographic variables, leisure time physical activity, smoking habits, chronic illnesses, respiratory symptoms, and medication were obtained from a questionnaire that was mailed to and answered by the participants before their visit to the research centre. Asthma was defined according to the ECRHS criteria [24] as a confirmatory answer to at least one of the following three questions ‘*Have you been woken by an attack of shortness of breath at any time during the last 12 months*?’, ‘*Have you had an attack of asthma within the last 12 months*?’ and ‘*Are you currently taking any medication (including inhalers, aerosols or tablets) for asthma*?’ Wheezing without a cold (in the following referred to as wheezing) was defined as a confirmatory answer to *‘Have you had wheezing or whistling in your chest at any time during the last 12 months?’* combined with a confirmatory answer to *‘If yes, have you had this wheezing or whistling when you did not have a cold?*’. Smoking was recorded as pack years (one pack year equalling one pack of cigarettes per day for a year) and categorised in five levels: (1) never smokers, (2) 0- ≤ 10, (3) 10- ≤ 30, (4) 30- ≤ 50, or (5) more than 50 pack years but was also used as a continuous measure. Educational level was categorised as: (1) less than 2 years, (2) skilled worker, (3) less than 3 years, (4) 3–4 years, and (5) more than four years. All questions used in the present analyses were identical in the baseline and follow-up studies.

### Physical measurements and assessment of lung function and F_E_NO

Anthropometric measures were obtained with participants in light clothing and without shoes. Height was measured to the nearest cm, weight to the nearest 0.1 kg, and body mass index (BMI) was calculated as kg/m^2^. Waist circumference (WC) was measured to the nearest cm using a tape measure midway between the lower rib margin and the iliac crest. Fat percentage was measured using a foot-to-foot Tanita Body Composition Analyzer (TBF-300, TANITA Corporation of America, Inc., Illinois, U.S.A.). Spirometry was performed according to American Thoracic Society and the European Respiratory Society (ATS/ERS) standard [25] using Spiro USB Spirometers (MicroMedical Limited, Rochester, Kent, UK). Spirometers were checked daily with a 3-liter calibrated syringe, and checked every six months with a decompression flow simulator [26]. F_E_NO measurements were performed only at baseline using a NIOX MINO (Aerocrine AB, Stockholm, Sweden). A dynamic flow restrictor yielded a constant flow rate of 50 mL/s, in accordance with recommendations of the ATS/ERS guidelines for F_E_NO measurement [27]. Nitric oxide concentrations between 5 and 300 ppb were measured and measurements below 5 ppb were set to zero by the NIOX MINO. Nitric oxide concentrations above 20 ppb were considered high.

### Biomarkers

Baseline serum samples were analysed for serum specific IgE against the four most clinically important inhalant allergens in Denmark: birch, grass, cat, and the house dust mite *Dermatophagoides pteronyssinus*, using the ADVIA Centaur Specific IgE assay [28]. Atopy was defined as at least one positive test (≥0.35 kilo units/L) against specific IgE. Plasma glucose concentrations were analysed by a hexokinase/glucose-6-phosphate dehydrogenase assay (Roche Diagnostic, Germany), and insulin concentrations were measured by fluoroimmunoassay technique (Dako Diagnostics Ltd., UK). The homeostatic model assessment-insulin resistance (HOMA-IR) index was calculated as: fasting plasma glucose (mmol/L) x fasting serum insulin (mU/L)/22.5 and applied only to the non-diabetic individuals. High-density lipoprotein (HDL) and triglyceride concentrations were measured by standard enzymatic techniques (Roche Diagnostic, Germany).

### Statistics

We used the R-statistical package, version 3.0.2 (http://www.r-project.org/) for all analyses. All p-values are two-tailed and statistical significance defined as p <0.05. P-values of likelihood ratio tests were used to test for significance of all multivariate analyses. We used linear regression and checked for equal residual variance across the full range of all exposure variables to check the linear model assumptions. We also tested possible non-linear associations by dividing the exposure variables in splines and by testing the quadratic term of the variables in the regression models. For our main analyses, we used absolute or percentage changes (between baseline and five-year follow up) of the adiposity measures to changes in FEV1 and FVC. The changes of FEV1 (or FVC) were modelled as five-year FEV1 (or FVC) adjusted for FEV1 (or FVC) at baseline. We also tested the ‘reverse’ association of baseline lung function measures to changes of adiposity measures. The adiposity measures were standardised by division of each measure by its own standard error to ease interpretation of the regression coefficients and to ease a comparison of the magnitude of effects between the different adiposity measures. In analyses of incidence of asthma and wheezing, logistic regression was used, similar model checks were applied, and the adiposity measures were categorised in four equally sized groups by quartiles of their respective distributions. Possible interactions were tested by adding an interaction term to the regression models, e.g. (adiposity measure*atopy) or (adiposity measure*gender). Finally, all analyses were repeated with s-Trig, s-HDL, and HOMA-IR included in the models to test whether these metabolic markers would attenuate the estimates found for adiposity. Sensitivity analyses included exclusion of the lower and upper 0.0025, 0.005, 0.01 fractiles of the adiposity measures, as well as separate analyses in non- and never smokers, and in younger or older (age categories were +/− 40, +/−45, +/−50 years) individuals.

Finally, we made a simulation-model based on randomly generated heights, BMI-levels, and levels of lung volume dependent on height. This model gives an example of how adipose tissue above a certain ‘person-dependent ideal’ would influence lung function (Additional file 1).

## Results

The baseline characteristics of the study population are given in Table 1. All characteristics are listed separately for participants and non-participants in the follow-up study. Of note is that non-participants as compared to participants had significantly higher levels of adiposity measures and higher levels of FEV1 and FVC. However, the baseline associations of adipose tissue levels with asthma, wheezing, FEV1 or FVC were not significantly different between participants and non-participants, all p-values for interaction between participation and each adiposity measure were between 0.12 and 0.98. Sensitivity analyses did not change the pattern of results presented.

**Table 1:** **Baseline characteristics of the study population according to participation in the five-year follow-up study**

	Participants follow-up			Non-participants follow-up		
	N = 2294			N = 1163		
	Mean	Sd	n miss	Mean	Sd	n miss
Age (y)	50.02	12.49		48.02	13.98	
FEV1 (liter)	3.16	0.79	9	3.04	0.88	5
FVC (liter)	4.03	0.96	9	3.89	1.06	5
	**N**	**% group**		**N**	**% group**	
*Gender*						
Males	1048	45.7		495	42.6	
*Adiposity measures*						
18.5 > BMI (kg/m2)	38	1.7		27	2.3	
18.5 < =BMI < 25 (kg/m2)	1096	47.8		491	42.2	
25 < =BMI < 30 (kg/m2)	839	36.6		408	35.1	
30 < =BMI (kg/m2)	319	13.9	2	237	20.4	
WC, normal^§^	1748	76.2		359	50.7	
WC, high^§^	545	23.8	2	349	49.3	3
*Serum lipids*						
HDL, normal^§^	2036	89.1	10	993	86.3	13
HDL, low^§^	249	10.9		158	13.7	
Triglyc <150 mg/dl	1901	83.2		887	77.1	
Triglyc 150–500 mg/dl	230	10.1		161	14.0	
Triglyc > = 500 mg/dl	154	6.7	10	103	8.9	13
*Atopy, asthma, wheezing*						
Atopy*, not present	1741	75.9		924	79.4	
Atopy*, present	553	24.1		239	20.6	
Asthma, not present	1872	82.2		906	78.9	
Asthma, present	405	17.8	17	242	21.1	15
Wheezing, not present	1974	86.7		915	79.9	
Wheezing, present	303	13.3	17	230	20.1	18
*Lifestyle*						
Smoking, never	1027	48.2	18	417	38.9	
Smoking, 0- ≤ 10 pack years	492	23.1		253	23.6	
Smoking, >10 pack years	613	28.8		403	37.6	89
Alcohol, no drinking	225	10.1		159	14.3	
Alcohol 1–14 units/week	1448	65.0		714	64.4	
Alcohol >14 units/week	555	24.9	66	236	21.3	54
Education^§^, none	239	10.5		215	18.8	
Education^§^, 1 year/practical	543	23.9		272	23.8	
Education^§§^, 2–3 years	943	41.4		450	39.3	
Education^§§^, > = 4 years	551	24.2	18	208	18.2	18

FEV1, forced expiratory volumes first second; FVC, forced vital capacity; BMI, body mass index; WC, waist circumference; Diabetes*, either self-reported diabetes or fasting p-glucose > =7.1 mmol/L; HDL, serum level of high-density lipoprotein; Triglyc, serum level of triglyceride; *Atopy defined as serum specific IgE positivity (> = .35 kU/l) to inhalant allergens; ^§^above primary school, ^§§^above high school.

**Figure 1:**
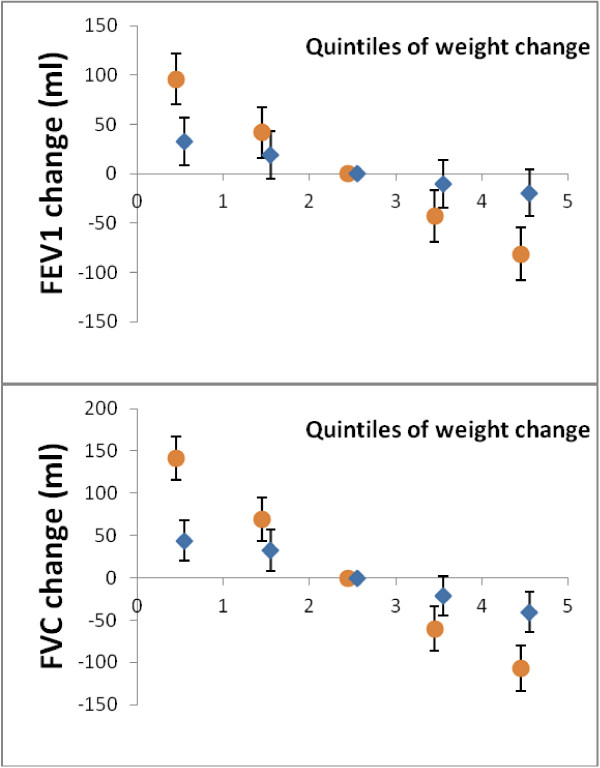
**Changes of lung function according to weight changes.** Five-year changes of FEV1 (upper panel) and FVC (lower panel) in quintiles of weight change in males (circles) and females (diamonds). Quintile one-two include individuals with weight loss, quintile four-five include individuals with weight gain. The middle quintile is used as reference. Analysis adjusted for age, tobacco use (pack years), and atopy.

From baseline to five-year follow-up, lung function was inversely associated with the level of adiposity as reflected by all four adiposity measures. In participants with weight gain, lung function declined; whereas in participants with weight loss, lung function increased to a similar extend (Figure 1 and Table 2). This pattern was observed in both males and females but the strength of these associations differed significantly between the two genders (Table 2): For all four adiposity measures, the estimated effect of adiposity changes on changes in FEV1 and FVC were approximately three times higher in males than in females in absolute numbers. The percentage changes were the double in males as compared to females. For instance, for every one standard deviation of weight change (5.4 kg) from baseline to follow-up, FEV1 either increased or decreased with approximately 72 ml (66-78 ml) in males and 26 ml (21-31 ml) in females, similarly FVC either increased or decreased with 111 ml (101-121 ml) in males and 35 ml (27-43 ml) in females. The metabolic markers, s-triglyceride, s-HDL, or HOMA-IR did not attenuate the effects of adiposity measures on changes in FEV1 and FVC (data not shown). Estimated mean declines for one year of increasing age were approximately 32.8 (31.3-34.4) ml FEV1 and 21.4 (19.0-23.9) ml FVC. In never-smokers, the corresponding estimates were 31.0 (28.7-33.3) ml FEV1and 21.0 (17.4-24.6) ml FVC. All age-estimates were essentially similar in males and females but were significantly smaller in younger than in older individuals.

**Table 2:** **Five-year changes of FEV (ml) and FVC (ml) in males and females per one standard deviation (1 sd) increase of adiposity measure**

	Males			Females			
	Est (ml)	Se (ml)	P	Est (ml)	Se (ml)	p	p-i
**FEV1**							
Weight (1 sd)	−72.6	5.8	<.001	−27.3	4.9	<.0001	<.001
BMI (1 sd)	−78.5	6.5	<.001	−25.5	4.6	<.0001	<.001
WC (1 sd)	−66.4	6.1	<.001	−23.4	4.8	<.0001	<.001
FatP (1 sd)	−52.0	6.2	<.001	−19.2	4.9	<.0001	<.001
**FVC**							
Weight (1 sd)	−103.2	9.0	<.001	−36.2	7.6	<.0001	<.001
BMI (1 sd)	−113.6	10.0	<.001	−32.6	7.1	<.0001	<.001
WC (1 sd)	−90.7	9.4	<.001	−28.1	7.4	<.0001	<.001
FatP (1 sd)	−54.3	9.7	<.001	−18.0	7.6	0.01	0.002

Linear regression models fitted with interaction between the specific adiposity measure and gender. Adjusted for age, atopy, tobacco use (pack years). Est, beta-estimate; Se, standard error for Est; p, p-value; p-i, p-value for difference (interaction) between males and females; BMI, body mass index; WC, waist circumference; FatP, fat percentage.

**Figure 2:**
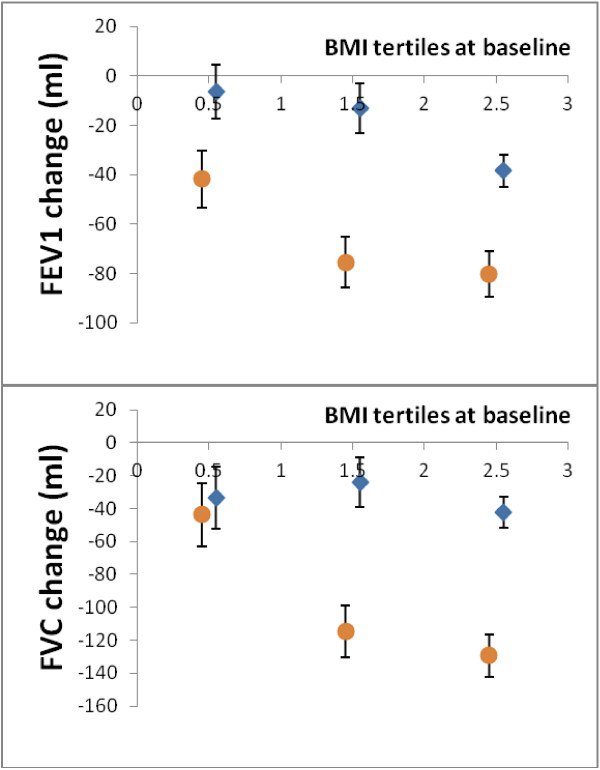
**Changes of lung function according to weight changes and baseline adiposity.** Five-year changes of FEV1 (upper panel) and FVC (lower panel) per one standard deviation increase of weight between baseline and follow-up according to baseline levels of BMI (X-axis) in males (circles) and females (diamonds), adjusted for age, atopy, tobacco use (pack years), and atopy.

The five-year adiposity changes had a differential impact on lung function changes according to baseline adiposity levels, as assessed as baseline BMI (Figure 2). Five-year adiposity changes were associated with dose-dependent greater declines of FEV1 and FVC in participants who were overweight and obese at baseline as compared to participants who were lean at baseline (Figure 2). Again, this effect was significantly different in males and females but the interaction of baseline-BMI*five-year changes of adiposity was only significant with respect to FVC (p = 0.02). Essentially similar results can be obtained by running our simulation example (Additional file 1). There were not any associations between changes of adiposity with changes of the FEV1/FVC-ratio between baseline and follow-up. For one standard deviation change of any adiposity measure, the FEV1/FVC-ratio changed between −0.0023 (fat percentage) to 0.00097 (BMI) in males (p-values 0.14-0.83) and this was not different in females (p for interactions 0.66-0.99).

To further investigate the temporal relationship of associations between changes in adiposity and lung function, we performed ‘reverse’ analyses where baseline FEV1 and FVC were used as explanatory variables for subsequent adiposity changes. However, there were no such statistically significant associations. Thus, these results support that changes in adiposity have an influence on lung function, but lung function does not have a similar influence on changes in adiposity.

The inverse association of adiposity with lung function was confirmed in both persons with and without asthma at baseline (Table 3). The gender differences in lung function declines from baseline to follow-up were also independent of asthma status at baseline (Table 3). With respect to atopy and F_E_NO -levels, there were essentially no differences in the associations of adiposity changes to changes of FEV1 and FVC between atopic and non-atopic individuals or between persons with or without elevated F_E_NO-levels (Table 4). Analyses in never- and non-smokers gave similar results (data not shown).

**Table 3:** **Five-year changes of FEV1 (ml) and FVC (ml) in males and females per one standard deviation increase of BMI from baseline to follow-up, according to asthma status at baseline**

		Males			Females		
		Est	Se	p	Est	Se	p
**FEV1**	Asthma	−96.4	16.7	<.001	−10.7	10.8	0.32
	Non-asthma	−75.6	7.0	<.001	−29.1	5.2	<.001
**FVC**	Asthma	−109.7	24.4	<.001	−15.0	15.8	0.34
	Non-asthma	−115.0	11.0	<.001	−37.3	8.1	<.001

All models fitted with BMI-gender interaction (all interactions significant, p ≤ .001) and adjusted for age, atopy, and smoking (pack years). BMI, body mass index; Est, beta-estimate; Se, standard error; ml, milliliter; p, p-value for Est.

**Table 4:** **Five-year changes of FEV (ml) and FVC (ml) in non-atopic and atopic individuals (upper panel), and in individuals with or without elevated FeNO-levels (lower panel), per one standard deviation increase of adiposity measures**

	Est (ml)	Se (ml)	P	Est (ml)	Se (ml)	P	p-i
	**Non-atopic**			**Atopic**			
**FEV1**							
Weight (1 sd)	−46.0	4.2	<.001	−46.2	8.5	<.001	ns
BMI (1 sd)	−42.3	4.2	<.001	−47.0	8.6	<.001	ns
WC (1 sd)	−38.9	4.3	<.001	−43.1	8.3	<.001	ns
FatP (1 sd)	−30.6	4.3	<.001	−35.8	8.5	<.001	ns
**FVC**							
Weight (1 sd)	−65.5	6.5	<.001	−59.3	13.1	<.001	ns
BMI (1 sd)	−61.6	6.6	<.001	−54.8	13.2	<.001	ns
WC (1 sd)	−53.2	6.6	<.001	−48.6	12.8	<.001	ns
Fat (1 sd)	−32.3	6.7	<.001	−32.1	13.1	<.02	ns
	**Normal FeNO (<=20 ppb)**			**Elevated FeNO (>20 ppb)**			
**FEV1**							
Weight (1 sd)	−45.6	4.7	<.001	−48.7	7.6	<.001	ns
BMI (1 sd)	−43.4	4.7	<.001	−43.9	7.7	<.001	ns
WC (1 sd)	−43.3	4.8	<.001	−31.7	7.2	<.001	ns
FatP (1 sd)	−31.8	4.8	<.001	−24.3	7.4	<.001	ns
**FVC**							
Weight (1 sd)	−57.1	7.2	<.001	−85.2	12.6	<.001	0.04
BMI (1 sd)	−54.5	7.2	<.001	−78.5	12.8	<.001	ns
WC (1 sd)	−48.1	7.5	<.001	−59.4	11.9	<.001	ns
FatP (1 sd)	−32.2	7.5	<.001	−28.1	12.4	<.02	ns

Linear regression models fitted with interaction between adiposity measure and either atopy or elevated FeNO and adjusted for age, atopy, and smoking (pack years); p-i, p-values for interaction test. Est, beta-estimate; Se, standard error of Est; p, p-value; Atopy defined as serum specific IgE positivity (> = .35 kU/l) to inhalant allergens; FeNO, fractional expiratory nitric oxide;1 sd, one standard deviation; ns, non-significant; BMI, body mass index; WC, waist circumference; FatP, fat percentage.

Finally, we investigated whether baseline levels of adiposity measures or changes of adiposity levels from baseline to follow-up were associated with incident asthma and wheezing. The incidence of asthma and wheezing between baseline and follow-up was 7.0% (158/2265) and 5.9% (133/2263), respectively. There were no statistically significant associations of any adiposity measure (baseline or changes) with incident asthma but for changes of weight and BMI there was a positive association with wheezing (Table 5). Notably, the novel cases of asthma in our cohort could be categorised as ‘late-onset’ asthma because all participants were > =19 years at baseline. Restricting our analysis further to novel cases of asthma and wheezing in participants e.g. above > = 25 years or > = 40 years yielded similar results. There were no differences between atopic and non-atopic individuals or individuals with or without elevated F_E_NO-levels with respect to incident asthma or wheezing (data not shown).

**Table 5:** **The association of changes of adiposity measures (quartiles) between baseline and five-year follow-up with risk of novel (incident) asthma and wheezing**

			Incident asthma					Incident wheezing				
		N	n	n/N	OR	CI1	CI2	n	n/N	OR	CI1	CI2
**Weight**	**Kg**											
q1	<−1.93	570	32	5.7	*ref*	-	-	26	4.7	*ref*	-	-
q2	1.93 - <0.7	573	47	8.3	1.47	0.91	2.42	27	4.7	0.89	0.48	1.63
q3	0.7 - <3.2	570	32	5.7	0.95	0.56	1.62	34	6.0	1.25	0.72	2.20
q4	>3.2	576	46	8.1	1.17	0.71	1.95	46	8.1	1.57	0.93	2.70
**BMI**	**Kg/m** ^**2**^											
q1	<−0.57	572	36	6.4	*ref*	-	-	25	4.5	*ref*	-	-
q2	−0.57 - <0.31	572	43	7.6	1.17	0.72	1.90	27	4.8	1.02	0.56	1.86
q3	0.31 - <1.216	572	36	6.4	1.00	0.61	1.65	35	6.2	1.35	0.77	2.41
q4	>1.22	573	42	7.4	0.88	0.53	1.43	46	7.1	1.69	0.99	2.94
**WC**	**Cm**											
q1	<−1.5	564	30	5.4	*Ref*	-	-	27	4.9	*ref*	-	-
q2	−1.5 - <2	576	39	6.9	1.15	0.68	1.96	25	4.4	0.85	0.46	1.57
q3	2 - <5	525	45	8.7	1.62	0.98	2.71	43	8.3	1.67	0.98	2.91
q4	>5	626	43	6.9	1.14	0.69	1.91	38	6.2	1.27	0.74	2.22
**FatP**	**Percentage points**											
q1	<−1.40	569	32	5.7	*ref*	-	-	31	5.0	*ref*	-	-
q2	−1.40 - <0.50	570	47	8.3	1.45	0.89	2.38	34	6.0	1.13	0.66	1.96
q3	0.50 - <2.402	569	42	7.5	1.24	0.75	2.06	25	4.5	0.77	0.42	1.38
q4	>2.40	570	33	5.8	0.78	0.45	1.35	40	7.1	1.20	0.71	2.05

Number of participants in each group (N), number of novel cases (n) and age, gender, and tobacco (pack years) adjusted odds ratios (OR) with 95% confidence intervals (CI1-CI2)); q1-q4, quartiles 1-4; BMI, body mass index; WC, waist circumference; FatP, Fat percentage.

## Discussion

We quantified the longitudinal relationship of several adiposity measures, including waist circumference and fat percentage, with lung function reflected by FEV1 and FVC. Thus, we confirmed changes of FEV1 and FVC per one kilo weight increase as summarised in Additional file 2 and extended that knowledge by reporting longitudinal estimates for abdominal adiposity (waist circumference) and fat percentage. We found that an increase in adiposity over five years was associated with declines in FEV1 and FVC, whereas a decrease in adiposity was associated with increases of FEV1 and FVC. These associations were more pronounced in males than in females but were independent of atopy and F_E_NO -levels. Adiposity changes were not associated with changes in the FEV1/FVC-ratio and were not associated with incident asthma.

Adipose tissue and especially upper body fat have in cross-sectional studies been shown to be associated with decreased static lung volumes as assessed by functional residual capacity and expiratory reserve volume [16, 17, 29]. As expiratory flow velocity is dependent on the degree of previous lung inflation due to the elastic properties of the lung [30], lower static lung volumes would subsequently impact on expiratory flows. Our results support such mechanical changes in a longitudinal setting. Firstly, the longitudinal adiposity changes were inversely associated with expiratory flow as reflected by FEV1. Secondly, pre-existing adiposity (at baseline) enhanced the negative impact of adiposity gain (Figure 2). Thus, the cumulative mass of adipose tissue was associated with reduced lung function as assessed by FEV1 and FVC. Adding that the FEV1/FVC-ratio remained unchanged, there was not a measurable increasing obstructive pattern with increasing adiposity. In comparison, inflammatory mechanisms would be expected to induce asthma-like, obstructive alterations in lung function [31].

The gender differences in the adiposity-lung function associations in this study were substantial, and may emphasise the mechanical aspects as males have more abdominal fat for the same degree of adiposity than females [32]. Similar conclusions have been put forward by authors in other studies [4, 33, 34] but supplementary explanations may exist. At least in asthma patients, recent cluster analyses have shown that a subgroup of non-adipose males have a high yearly loss of FEV1 that is not explained by presence or absence of atopy, the duration of disease etc. [35, 36]. This could indicate that another pathological mechanism towards declines of lung volumes occurs in those males, and this could also apply to males without asthma. Differences in sex hormonal levels, which are emphasised by decreasing gender differences in lung volume decline in elderly [3] is a possible explanation for an apparently different airway pathology in males and females. Other possibilities are different thresholds for the detrimental effects of pulmonary irritants, or differences in airway calibre between males and females [37], although none have been fully documented so far.

Atopy or F_E_NO-levels did not modify the effects of adiposity on lung function. Thus, an underlying atopic as compared to non-atopic condition or an underlying eosinophilic as compared to non-eosinophilic airway inflammation did not appear to enhance the inverse association of adiposity with lung function in our general population. It is possible that a characterisation of participants by e.g. eosinophils in sputum or blood would have yielded other results, as elevated FENO-levels only are indicative of eosinophil airway inflammation [38]. For instance, cluster analyses have indicated that some groups of asthma patients are characterised by eosinophil airway inflammation and low lung function [39]. Further, faster declines of lung volumes in non-atopic as compared to atopic asthma patients have also been reported [36, 40, 41]. However, our study was conducted in a general population and not in specific groups of patients. Thus, absence of effect modification by atopy or F_E_NO-levels in our study appears to emphasise restrictive changes of lung function with increasing adiposity. However, our study was not powered to investigate effect modifications. Therefore, more studies are needed to confirm or refute the presence of effect modification by atopy or elevated F_E_NO-levels.

We did not find an association between increasing adiposity and asthma incidence but for increasing weight and BMI there was a positive association with incident wheezing. This is in contrast with findings of most longitudinal studies, which have reported a positive association between increasing BMI and incidence of asthma [1]. However, the power to detect such differences in the present study was relatively low as indicated by relatively wide 95% confidence intervals. Possibly, testing the association of adiposity with bronchial hyperreactivity (BHR) would have yielded other results; in a previous study we found a positive association of adiposity with BHR [42], but such information was not available in the current study.

The strengths of the study include the prospective design, validated and similar methods for measurements of FEV1 and FVC at baseline and follow-up, assessment of atopy by serum specific IgE and airway eosinophil inflammation by F_E_NO. The limitations include the lack of tests for variability of lung function to define asthma, lack of measures of lung volumes, and that the analyses are based on the assumption that increases or decreases of weight and BMI were due to adipose tissue and not muscle mass. Selection bias may have decreased the generalisability of the study and loss to follow-up influenced the reported associations. However, we found similar baseline associations of adipose tissue measures with all outcomes in participants and non-participants in the follow-up indicating that our results may apply to a general population.

## Conclusion

In conclusion, changes of adiposity had a high, gender specific impact on FEV1 and FVC but not on the FEV1/FVC-ratio. Increasing adiposity was associated with lung function decline, whereas loss of adiposity was associated with increasing lung function. These associations were independent of atopy, F_E_NO -levels, and metabolic markers, but were modified by the level of adipose tissue prior to the adiposity changes indicating the importance of aggregate adipose tissue mass. The adiposity changes leading to declines of FEV1 and FVC were not concomitantly associated with incidence of asthma or wheezing, however, a low number of novel cases of asthma and wheezing may have limited the power to detect associations with these outcomes.

## Electronic supplementary material

Additional file 1: **Simulation model of lung function depending on BMI.** (DOCX 15 KB)

Additional file 2: **Overview of longitudinal studies of the association between increasing adiposity and decreasing lung function conducted in general adult populations.** (DOCX 24 KB)

## References

[1] Beuther DA, Sutherland ER (2007). Overweight, obesity, and incident asthma: a meta-analysis of prospective epidemiologic studies. Am J Respir Crit Care Med.

[2] Chinn S, Jarvis D, Melotti R, Luczynska C, Ackermann-Liebrich U, Anto JM, Cerveri I, de MR, Gislason T, Heinrich J, Janson C, Kunzli N, Leynaert B, Neukirch F, Schouten J, Sunyer J, Svanes C, Vermeire P, Wjst M, Burney P (2005). Smoking cessation, lung function, and weight gain: a follow-up study. Lancet.

[3] Rossi A, Fantin F, Di FV, Guariento S, Giuliano K, Fontana G, Micciolo R, Solerte SB, Bosello O, Zamboni M (2008). Body composition and pulmonary function in the elderly: a 7-year longitudinal study. Int J Obes (Lond).

[4] Carey IM, Cook DG, Strachan DP (1999). The effects of adiposity and weight change on forced expiratory volume decline in a longitudinal study of adults. Int J Obes Relat Metab Disord.

[5] Chinn DJ, Cotes JE, Reed JW (1996). Longitudinal effects of change in body mass on measurements of ventilatory capacity. Thorax.

[6] Wannamethee SG, Shaper AG, Whincup PH (2005). Body fat distribution, body composition, and respiratory function in elderly men. Am J Clin Nutr.

[7] Sutherland ER (2014). Linking obesity and asthma. Ann N Y Acad Sci.

[8] Mosen DM, Schatz M, Magid DJ, Camargo CA (2008). The relationship between obesity and asthma severity and control in adults. J Allergy Clin Immunol.

[9] Kronander UN, Falkenberg M, Zetterstrom O (2004). Prevalence and incidence of asthma related to waist circumference and BMI in a Swedish community sample. Respir Med.

[10] Appleton SL, Adams RJ, Wilson DH, Taylor AW, Ruffin RE (2006). Central obesity is associated with nonatopic but not atopic asthma in a representative population sample. J Allergy Clin Immunol.

[11] Granell R, Henderson AJ, Evans DM, Smith GD, Ness AR, Lewis S, Palmer TM, Sterne JA (2014). Effects of BMI, fat mass, and lean mass on asthma in childhood: a Mendelian randomization study. PLoS Med.

[12] Ronmark E, Andersson C, Nystrom L, Forsberg B, Jarvholm B, Lundback B (2005). Obesity increases the risk of incident asthma among adults. Eur Respir J.

[13] Haldar P, Pavord ID, Shaw DE, Berry MA, Thomas M, Brightling CE, Wardlaw AJ, Green RH (2008). Cluster analysis and clinical asthma phenotypes. Am J Respir Crit Care Med.

[14] Scott HA, Gibson PG, Garg ML, Wood LG (2011). Airway inflammation is augmented by obesity and fatty acids in asthma. Eur Respir J.

[15] Salome CM, King GG, Berend N (2010). Physiology of obesity and effects on lung function. J Appl Physiol (1985).

[16] Steier J, Lunt A, Hart N, Polkey MI, Moxham J (2014). Observational study of the effect of obesity on lung volumes. Thorax.

[17] Jones RL, Nzekwu MM (2006). The effects of body mass index on lung volumes. Chest.

[18] Wehrmeister FC, Menezes AM, Muniz LC, Martinez-Mesa J, Domingues MR, Horta BL (2012). Waist circumference and pulmonary function: a systematic review and meta-analysis. Syst Rev.

[19] Thyagarajan B, Jacobs DR, Apostol GG, Smith LJ, Jensen RL, Crapo RO, Barr RG, Lewis CE, Williams OD (2008). Longitudinal association of body mass index with lung function: the CARDIA study. Respir Res.

[20] Bottai M, Pistelli F, Di PF, Carrozzi L, Baldacci S, Matteelli G, Scognamiglio A, Viegi G (2002). Longitudinal changes of body mass index, spirometry and diffusion in a general population. Eur Respir J.

[21] Pistelli F, Bottai M, Carrozzi L, Pede FD, Baldacci S, Maio S, Brusasco V, Pellegrino R, Viegi G (2008). Changes in obesity status and lung function decline in a general population sample. Respir Med.

[22] Chen Y, Horne SL, Dosman JA (1993). Body weight and weight gain related to pulmonary function decline in adults: a six year follow up study. Thorax.

[23] Thuesen BH, Cerqueira C, Aadahl M, Ebstrup JF, Toft U, Thyssen JP, Fenger RV, Hersoug LG, Elberling J, Pedersen O, Hansen T, Johansen JD, Jorgensen T, Linneberg A (2014). Cohort profile: the health2006 cohort, research centre for prevention and health. Int J Epidemiol.

[24] Berg ND, Husemoen LL, Thuesen BH, Hersoug LG, Elberling J, Thyssen JP, Carlsen BC, Johansen JD, Menne T, Bonnelykke K, Stender S, Meldgaard M, Szecsi PB, Linneberg A (2012). Interaction between filaggrin null mutations and tobacco smoking in relation to asthma. J Allergy Clin Immunol.

[25] Miller MR, Hankinson J, Brusasco V, Burgos F, Casaburi R, Coates A, Crapo R, Enright P, van der Grinten CP, Gustafsson P, Jensen R, Johnson DC, Macintyre N, McKay R, Navajas D, Pedersen OF, Pellegrino R, Viegi G, Wanger J (2005). Standardisation of spirometry. Eur Respir J.

[26] Pedersen OF, Naeraa N, Lyager S, Hilberg C, Larsen L (1983). A device for evaluation of flow recording equipment. Bull Eur Physiopathol Respir.

[27] **ATS/ERS recommendations for standardized procedures for the online and offline measurement of exhaled lower respiratory nitric oxide and nasal nitric oxide, 2005** *Am J Respir Crit Care Med* 2005, **171:**912–930. 10.1164/rccm.200406-710ST 15817806

[28] Petersen AB, Gudmann P, Milvang-Gronager P, Morkeberg R, Bogestrand S, Linneberg A, Johansen N (2004). Performance evaluation of a specific IgE assay developed for the ADVIA centaur immunoassay system. Clin Biochem.

[29] Sutherland TJ, Goulding A, Grant AM, Cowan JO, Williamson A, Williams SM, Skinner MA, Taylor DR (2008). The effect of adiposity measured by dual-energy X-ray absorptiometry on lung function. Eur Respir J.

[30] Hayes D, Kraman SS (2009). The physiologic basis of spirometry. Respir Care.

[31] Shore SA (2010). Obesity, airway hyperresponsiveness, and inflammation. J Appl Physiol.

[32] Bjorntorp P (1991). Adipose tissue distribution and function. Int J Obes.

[33] Chen Y, Rennie D, Cormier YF, Dosman J (2007). Waist circumference is associated with pulmonary function in normal-weight, overweight, and obese subjects. Am J Clin Nutr.

[34] Leone N, Courbon D, Thomas F, Bean K, Jego B, Leynaert B, Guize L, Zureik M (2009). Lung function impairment and metabolic syndrome: the critical role of abdominal obesity. Am J Respir Crit Care Med.

[35] Moore WC, Meyers DA, Wenzel SE, Teague WG, Li H, Li X, D’Agostino R, Castro M, Curran-Everett D, Fitzpatrick AM, Gaston B, Jarjour NN, Sorkness R, Calhoun WJ, Chung KF, Comhair SA, Dweik RA, Israel E, Peters SP, Busse WW, Erzurum SC, Bleecker ER (2010). Identification of asthma phenotypes using cluster analysis in the severe asthma research program. Am J Respir Crit Care Med.

[36] Amelink M, de Nijs SB, Berger M, Weersink EJ, Ten BA, Sterk PJ, Bel EH (2012). Non-atopic males with adult onset asthma are at risk of persistent airflow limitation. Clin Exp Allergy.

[37] Chen Y, Rennie DC, Pahwa P, Dosman JA (2012). Pulmonary function in adults with recent and former asthma and the role of sex and atopy. BMC Pulm Med.

[38] Dweik RA, Boggs PB, Erzurum SC, Irvin CG, Leigh MW, Lundberg JO, Olin AC, Plummer AL, Taylor DR (2011). An official ATS clinical practice guideline: interpretation of exhaled nitric oxide levels (FENO) for clinical applications. Am J Respir Crit Care Med.

[39] Wu W, Bleecker E, Moore W, Busse WW, Castro M, Chung KF, Calhoun WJ, Erzurum S, Gaston B, Israel E, Curran-Everett D, Wenzel SE (2014). Unsupervised phenotyping of severe asthma research program participants using expanded lung data. J Allergy Clin Immunol.

[40] Marcon A, Corsico A, Cazzoletti L, Bugiani M, Accordini S, Almar E, Cerveri I, Gislason D, Gulsvik A, Janson C, Jarvis D, Martinez-Moratalla J, Pin I, de MR (2009). Body mass index, weight gain, and other determinants of lung function decline in adult asthma. J Allergy Clin Immunol.

[41] Ulrik CS, Backer V, Dirksen A (1992). A 10 year follow up of 180 adults with bronchial asthma: factors important for the decline in lung function. Thorax.

[42] Fenger RV, Linneberg A, Vidal C, Vizcaino L, Husemoen LL, Aadahl M, Gonzalez-Quintela A (2012). Determinants of serum tryptase in a general population: the relationship of serum tryptase to obesity and asthma. Int Arch Allergy Immunol.

[CR43] The pre-publication history for this paper can be accessed here:http://www.biomedcentral.com/1471-2466/14/208/prepub

