# Supplementary material for: The longitudinal relationship of changes of adiposity to changes in pulmonary function and risk of asthma in a general adult population
Source: BMC Pulm Med. 2014 Dec 22;14:208. doi: 10.1186/1471-2466-14-208 (PMC4364582; doi:10.1186/1471-2466-14-208)
Supplement: Supplementary file 1 — Additional file 1: Simulation model of lung function depending on BMI. (DOCX 15 KB) [file 12890_2014_643_MOESM1_ESM.docx]

**## Additional File 1**

# choosing number of participants

N <- 2300

# If the number of participants is low, it is important to run the script several times

# Three time points are used:

# t=0, participants reaching adulthood

# t=1, resembles baseline of the Health2006-study

# t=2, resembles follow-up of the Health2006-study

# Assumptions:

# BMI at t=0 was 'ideal'

# Positive BMI changes t=1 to t=2: the model assumes decreasing lung volumes

# Negative BMI changes t=1 to t=2: the model assumes somewhat increasing lung volumes

# as long as BMI does not decrease below the 'ideal' BMI at t=0

# Lung volumes depend on height of the participants

# time = 0, reaching adulthood

# generating BMI assuming normal distribution around BMI=21 kg/m2 with an additional a random error of 1 kg/m2

# This BMI resembles the 'ideal' body composition which differs from person to person

BMI0 <- rnorm(N,21,1)

# generating Height of participants assuming normal distribution around 1.75 metres with an additional random error of 0.1 cm

H <- rnorm(N,1.75,0.1)

# generating Lung volumes (L0)assuming dependency on heights and normal distribution

L0 <- 3*sqrt(H)+rnorm(N,0,0.3) #litres

# time = 1, "baseline"

# generating baseline-BMI (BMI1) assuming normal distribution of BMI changes but that mean BMI increased slightly (0.3 kg/m2).

# including a normally distributed error of 1 kg/m2

BMI1 <- BMI0+rnorm(N,0.3,1)

# generating lung volumes at time=1 (L1) assuming dependency on BMI and including a normally distributed error of 0.1 litres on L1

L1 <- L0-0.08*pmax((BMI1-BMI0),0)+rnorm(N,0,0.1)

# time = 2, "follow-up"

# generating BMI at follow-up (BMI2), similar assumptions as for BMI1

BMI2 <- BMI1+rnorm(N,0.3,1)

# generating lung volumes at time=2 (L2), similar assumptions as for L1

L2 <- L0-0.08*pmax((BMI2-BMI0),0)+rnorm(N,0,0.1)

# From above changes of BMI are found like:

deltaBMI <- BMI2-BMI1

# Making 1-4 variable of baseline BMI describing the quantile

quantiles <-quantile(BMI1)

quantiles[1] <- -1e-10

quantiles[length(quantiles)] <- 1e10

qBMI1 <- rep(NA,length(BMI1))

for(i in 2:length(quantiles))

{

qBMI1[quantiles[i-1]<=BMI1 & BMI1<quantiles[i]] <- i-1

}

qBMI1 <- factor(qBMI1)

## Analyses resembling those presented in the tables / Figure 1

# baseline cross-sectional model

fit <- glm(L1 ~ BMI1)

summary(fit)

# Changes of BMI as explanatory for lung volume changes

fit <- glm(L2 ~ deltaBMI + L1)

summary(fit)

# model with interaction: Changes of BMI interaction with baseline BMI-levels as explanatory for lung volume changes

fit <- glm(L2 ~ deltaBMI*qBMI1 + L1)

summary(fit)
