# Supplementary material for: The longitudinal relationship of changes of adiposity to changes in pulmonary function and risk of asthma in a general adult population
Source: BMC Pulm Med. 2014 Dec 22;14:208. doi: 10.1186/1471-2466-14-208 (PMC4364582; doi:10.1186/1471-2466-14-208)
Supplement: Supplementary file 2 — Additional file 2: Overview of longitudinal studies of the association between increasing adiposity and decreasing lung function conducted in general adult populations. (DOCX 24 KB) [file 12890_2014_643_MOESM2_ESM.docx]

| **Additional file 2. Overview of longitudinal studies of the association between increasing adiposity and decreasing lung function conducted in general adult populations** | | | | | | | | | | |
| --- | --- | --- | --- | --- | --- | --- | --- | --- | --- | --- |
|  | **Exam.** | **N** | **Age†** | **yrs^¶^** | **∆Adiposity** | **∆FEV1 (95%CI)/ml** | | **∆FVC (95%CI)/ml** | | **Adjustments** |
| Study | year |  |  |  |  | Males | Females | Males | Females |  |
| Chinn [2]  (ECRHS) | 1991-93 | 6654 | 20-44 | 7 y | 1 kg | -11.5  -(9.6;13.3) | -3.7  -(2.5;5.0) | -12.1  -(9.7;14.6) | -3.1  -(1.5;5.8) | Centre, age, height, smoking, mid-survey weight |
| Thyagarajan [19] | 1985-86 | 3950 | 18-30 | 10y | 1-2.4 kg | M+F**‡**: -30 (16;44) to  -81 (64;97) | | M+F**‡**: +53 +(38-67) to  -63 (46;80) | | Age, time of invest., race, sex, height, smoking, physical activity, prevalence and incidence of asthma |
| Carey [4] | 1984-85 | 3391 | 18-73 | 7 y | 1 kg | -9.6  -(6.5;12.7) | -5.1  -(2.7;7.5) | - | - | Region, six socio-economic groups, smoking pack-years, mid-survey weight |
|  |  |  |  |  | 1 cm, waist circumference | -8.8¶¶  -(6.6;10.9) | -4.7¶¶  -(3.0;6.4) |  |  |  |
| **Health2006** | **2006-08** | **2294** | **19-71** | **5 y** | **1 kg** | **-13.3**  **-(12.2**;**14.4)** | **-4.7**  **-(3.8-5.7)** | **-20.5**  **-(18.7**;**22.3)** | **-6.4**  **-(5.0**;**7.9)** | **Age, smoking, atopy** |
|  |  |  |  |  | **1 cm, waist circumference** | **-11.0**  **-(10.0:12.0)** | **-3.5**  **-(2.6;4.3)** | **-16.7**  **-(15.0-18.4)** | **-4.8**  **-(3.5;6.2)** |  |
| Pistelli/Bottai [20, 21] | 1980-82 | 1212 | >24 | 8 y | Becoming vs. never obese | M+F: -111 -(95;127) | | M+F: -122 -(104;146) | | Age, gender, smoking |
| Chen [22] | 1977 | 709 | 25-69 | 6 y | 1 kg | -23  -(3;45) | -9  -(5;13) | -26  -(17;35) | -14  -(9;18) | Age, smoking |
| Rossi [3] | ? | 77 | 72 (2) |  | 1 cm, *ASD | M+F: -31 -(15;46) | | M+F: -46 -(25;67) | | Age, gender, smoking,  s-albumine |
| Exam.year, the time of baseline study; N, number of participants in the study; † Age of participants at the beginning of the study as range or mean(sd); **^¶^** Follow-up length (years); **∆**Adiposity, change of adiposity; ∆FEV1, change of forced expiratory volume first second; ∆FVC, change of forced vital capacity; CI, confidence interval; ml, milliliters; M+F, result reported for males and females grouped together **‡** result depends on the level of previous adiposity, ¶¶ The results were different for different age groups, in the table the maximal changes reported in the study are displayed; *Additional sagittal diameter measured by DEXA-scan | | | | | | | | | | |
